# Application of RNA subcellular fraction estimation method to explore RNA localization regulation

**DOI:** 10.1093/g3journal/jkab371

**Published:** 2021-11-13

**Authors:** Xiaomin Dai, Yangmengjie Li, Weizhen Liu, Xiuqi Pan, Chenyue Guo, Xiaojing Zhao, Jingwen Lv, Haixin Lei, Liye Zhang

**Affiliations:** 1 School of Life Science and Technology, ShanghaiTech University, Shanghai 201210, China; 2 CAS Center for Excellence in Molecular Cell Science, Shanghai Institute of Biochemistry and Cell Biology, Chinese Academy of Sciences, Shanghai 200031, China; 3 University of Chinese Academy of Sciences, Beijing 100049, China; 4 Institute of Cancer Stem Cell, Cancer Center, Dalian Medical University, Dalian 116044, China

**Keywords:** RNA localization, nuclear retention, RBP, subcellular RNA abundance, *cis*-elements, isoform

## Abstract

RNA localization is involved in multiple biological processes. Recent advances in subcellular fractionation-based sequencing approaches uncovered localization pattern on a global scale. Most of existing methods adopt relative localization ratios (such as ratios of separately normalized transcripts per millions of different subcellular fractions without considering the difference in total RNA abundances in different fractions), however, absolute ratios may yield different results on the preference to different cellular compartment. Experimentally, adding external Spike-in RNAs to different fractionation can be used to obtain absolute ratios. In addition, a spike-in independent computational approach based on multiple linear regression model can also be used. However, currently, no custom tool is available. To solve this problem, we developed a method called subcellular fraction abundance estimator to correctly estimate relative RNA abundances of different subcellular fractionations. The ratios estimated by our method were consistent with existing reports. By applying the estimated ratios for different fractions, we explored the RNA localization pattern in cell lines and also predicted RBP motifs that were associated with different localization patterns. In addition, we showed that different isoforms of same genes could exhibit distinct localization patterns. To conclude, we believed our tool will facilitate future subcellular fractionation-related sequencing study to explore the function of RNA localization in various biological problems.

## Introduction

RNA localization plays important functions in diverse biological processes in the cells. Proper subcellular distribution of RNAs not only maintains the correct organization of cell structure but also is crucial to control multiple biological processes (Holt and Bullock 2009; Medioni *et al.* 2012; Holt and Schuman 2013; Bovaird *et al.* 2018). mRNAs localized to specific compartments in the cells allow proteins to be produced rapidly and energy-efficiently on-site rather than be transported with lag and cost especially in response to extrinsic stimuli (Martin and Ephrussi 2009; Medioni *et al.* 2012). For noncoding RNA, since noncoding RNAs broadly involved in multisteps of gene expression control in distinct localizations in the cell, proper partitioning of regulatory RNAs would be critical to promote interaction with targets and ensure efficient execution of their biological functions (Batista and Chang 2013). 

RNA localization involves multiple regulatory processes, such as RNA nuclear retention, cytosolic export, and transportation to specific compartments. *Cis*-elements encoded on RNAs are recognized by RBP and guide the RNA to the proper cellular localization of RNAs (Miyagawa *et al.* 2012; Chaudhuri *et al.* 2020). Many neuronal and oocyte mRNAs have extended 3’UTRs that contain the *cis*-elements dictating their specific localization pattern in the cells, which is referred to as RNA *cis*-acting zipcode elements (Jambhekar and Derisi 2007; Taliaferro *et al.* 2016; Tushev *et al.* 2018). However, *cis*-elements are not restricted to 3’ UTR but can be located at other exonic regions. For example, binding of HNRNPK to C-rich motifs outside Alu elements is associated with increased nuclear accumulation in both lncRNAs and mRNAs (Lubelsky and Ulitsky 2018).

Traditionally, RNA localization was studied on a gene-by-gene manner. Recently, the omics approaches were used to reveal the genome-wide localization patterns. A number of experimental approaches were available to perform genome-wide profiling for subcellular fractionations, such as subcellular RNA-Seq and CeFra-Seq (Djebali *et al.* 2012; Lefebvre *et al.* 2017). When performing the quantification and downstream analyses, most used relative localization ratios of separately normalized expression quantification (such TPM or FPKM values) without adjusting based on their absolute abundances in different fractions (Carlevaro-Fita and Johnson 2019). However, subcellular distribution of RNA molecules is asymmetric in the nuclear and cytosolic compartments: cytosol generally has a higher total RNA abundance than nucleus (Abdelmoez *et al.* 2018; Guo *et al.* 2020). A previous study adopted a multiple linear regression approach to estimate absolute abundances for lncRNA and mRNA in nuclear and cytosolic fractions separately (Carlevaro-Fita and Johnson 2019). Moreover, adopting relative and absolute ratios might yield different conclusions, thus suggesting the need to consider absolute ratios (Carlevaro-Fita and Johnson 2019). However, no available tool was provided for their method.

In this study, we provided a method and easy-to-use Python scripts to infer the relative abundance of different subcellular fractionations. Instead of estimating the absolute ratios for lncRNA and mRNAs separately adopted in multi linear regression method, we estimated only one single fraction ratio. The relative abundance of different cellular fractions predicted based on our method was consistent with existing results. Then we explored whether such relative abundance varied in different conditions. In addition to global level regulation, we also looked into the distribution pattern for individual genes in these conditions. Finally, we tried to explore the regulatory mechanism and potential functional significance of the variations of RNA localization.

## Materials and methods

### Data preparation

The processed gene and transcript TPM expression level data of subcellular polyA and non-PolyA samples were downloaded from ENCODE database (https://www.encodeproject.org/). There are 11 cell lines with Cytosol, Nucleus and whole cell (WC) fractions PolyA RNA-seq, 7 cell lines with RNA-seq from three corresponding non-PolyA fractions (Tilgner *et al.* 2012). The detailed sample information could be found in Supplementary Table S1.

### RNA-seq data preprocessing

The RNA-seq data about HNRNPK was downloaded from the SRA database, accession SRP111756 (Lubelsky and Ulitsky 2018). The sequenced reads were mapped to human reference genome (GRCh38.89) using the STAR (STAR_2.5.3a) mapping program with parameters recommended by ENCODE project (Dobin *et al.* 2013). The expression TPM and FPKM of genes and isoforms were calculated by Expectation-Maximization (RSEM v1.3.0; Li and Dewey 2011).

### Subcellular fraction abundance estimator method to estimate the cytosolic ratios

The inputs of subcellular fraction abundance estimator (SFAE) method were the filtered normalized expression vectors: genes whose TPM level in WC did not fall within the range between nuclear and cytosol TPM levels were removed. To estimate the single parameter CR, we aimed to identify CR value that minimized the difference between the predicted WC TPM vector with observed TPM vector, where TPM_predicted_ is equal to CR*TPM_cyto_ + (1-CR)*TPM_nuc_. The cost function we used to minimize the differences is

$$E\left(\theta \right)=\frac{\sum_{i=1}^{n}{(\ln\frac{\theta \cdot {\mathrm{TPM}}_{\mathrm{Cyto},\;i}+（1-\theta ）\cdot {\mathrm{TPM}}_{\mathrm{Nuc},\;i}}{{\mathrm{TPM}}_{WC,i}})}^{2}}{n},$$

where θ represented cytosolic RNA abundance ratio (CR), ${\mathrm{TPM}}_{\mathrm{Cyto},\;i}$, ${\mathrm{TPM}}_{\mathrm{Nuc},\;i}$, and ${\mathrm{TPM}}_{WC,i}\;$was corresponding to TPM value of gene i in Cytosol, Nucleus, and WC fractions, respectively, and n was the gene number involved in estimation after filtering in preprocessing step. We used constrained minimization method “trust-constr” implemented in scipy.optimize.minimize function in Scipy package in Python to estimate the CR ratios (Virtanen *et al.* 2020).

**Figure 1:**
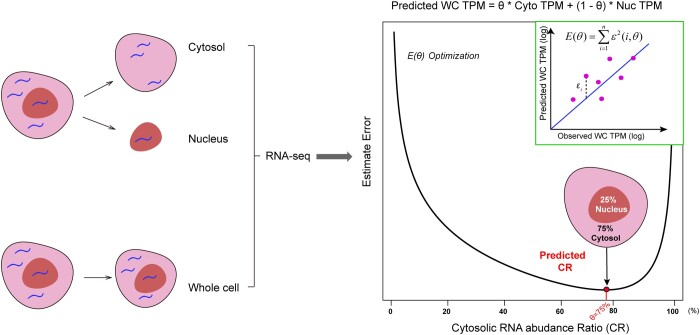
Overview of pipeline. Cells are separated into cytosol and nucleus fractions, then Cytosol, Nucleus, and WC fractions are separately sequenced (left). With processed TPM data of three fractions, true CR could be calculated with SFAE as figure shows (right); *E(θ)* is defined as summed estimated error between predicted- and observed-TPM data of WC for all genes. For each CR or *θ* in this example, an Estimate Error between predicted- and observed-TPM data of WC was calculated. Then, the CR value with the minimum estimated error (red point) is considered to be optimal estimate of CR.

We’ve tested in multiple datasets and such cost function would normally yield a bell shape in the prediction error measurement (Figure  1). When dealing subcellular fractionation with more than two fractions, similar ratios for each fraction can be estimated simultaneously with the same function. The method is available as a standalone Python pipeline (github.com/bioliyezhang/SFAE).

### Cell culture and actinomycin D treatment

Hela cell were gifts from Reed Lab in Harvard Medical School, All the cells were cultured in DMEM medium supplemented with 10% FBS(Gibco), 1% streptomycin/penicillin and 0.1% gentamycin(Life Technologies). Cell were incubated at 37°C and 5% CO_2_. HeLa cells were treated with 5 µg/ml actinomycin D (ActD; Merck) to inhibit transcription (Hou *et al.* 2019), DMSO was added as a control (5%), and then harvested at the indicated time points following addition of ActD.

### Nuclear and cytoplasmic separation, RNA isolation, and Library Prep

For separation of nuclear and cytoplasmic fractions, the extraction kit (Beyotime China, P0027) was used and followed the same procedures as previous study (Khan *et al.* 2021). Total RNA was used as input material for the RNA sample preparations. Briefly, mRNA was purified from total RNA by using poly-T oligo-attached magnetic beads. Fragmentation was carried out using divalent cations under elevated temperature in First Strand Synthesis Reaction Buffer (5X). First strand cDNA was synthesized using random hexamer primer and M-MuLV Reverse Transcriptase, then use RNaseH to degrade the RNA. Second strand cDNA synthesis was subsequently performed using DNA Polymerase I and dNTP. Remaining overhangs were converted into blunt ends via exonuclease/polymerase activities. After adenylation of 3’ ends of DNA fragments, Adaptor with hairpin loop structure were ligated to prepare for hybridization. In order to select cDNA fragments of preferentially 370–420 bp in length, the library fragments were purified with AMPure XP system (Beckman Coulter, Beverly, USA).

### Motif enrichment analysis for the three genesets in human

We extracted cDNA sequence of the most highly expressed isoform (defined by the isoform with the highest total sum of expression TPM values) across multiple cell lines for each gene in three genesets from ensemble reference database (Homo_sapiens.GRCh38.cdna.all.fa; Mus_musculus.GRCm38.cdna.all.fa). Then we used FIMO to scan each gene’s cDNA sequence for occurrences of the annotated motifs from CISBP-RNA and Ray 2013 motif databases from MEME website based on default setting (Grant *et al.* 2011). To remove the bias driven by gene length, we calculated the motif density (normalized by gene length) for each gene given a RBP motif. Then we used Wilcoxon rank-sum test to examine whether the motif densities (motif occurrences divided by most abundant isoform length) were significantly different between genes in one geneset *vs* the rest of genes. Then *P*-values were then corrected for multiple hypotheses testing by FDR method.

For genesets of mouse, we directly used the genesets (Cyto-gene and Nuc-gene) defined in their study (Halpern *et al.* 2015). Cytoplasmic genes (Cyto-genes) are genes whose log2(Number_*Cyto*_/Number_*Nuc*_) more than 0 in both MIN6 cells and liver cells (Number_*Cyto*_ and Number*_Nuc_* respectively represent normalized numbers of cytoplasmic and nuclear mRNAs per cell in MIN6 cells and liver cells). Similarly, nuclear genes (Nuc-genes) are genes whose log2 (Number_*Cyto*_/Number_*Nuc*_) less than 0 in both MIN6 and liver cells.

### Identification of isoforms that are significantly correlated with CRs values

First, correlation between CR of each gene and max/second expressed transcript were calculated using Pearson correlation coefficient. Second, the genes whose absolute correlations of the most and second-most abundantly expressed transcripts are both more than 0.4 and median TPM of genes are greater than 3 were chosen. Finally, 335 genes with opposite correlation pattern (positive and negative correlation) in two isoforms were remained.

## Results

### Estimation of relative RNA abundance for subcellular compartment

We used *θ_i_* (ranges from 0 to 1) to represent the ratios of total RNA abundances among individual cellular subfractions. When cells are fractioned into nuclear and cytosolic portions, we used *θ_cyto_* to represent the CR. For each fraction as well as total cell extract, we have a vector of transcripts per million (TPM) values (such as TPM_Cyto_, TPM_Nuc_, and TPM_WC_) to represent the expression vectors. We estimated optimal *θ_Cyto_* by minimizing the sum of log ratios of the observed and predicted expression matrix (Figure  1 and see more details in *Materials and* *Methods* section). Such strategies can be also generalized to cases with more than two subcellular fractionations of cells, such as data from CeFra-Seq with four fractions.

**Figure 2:**
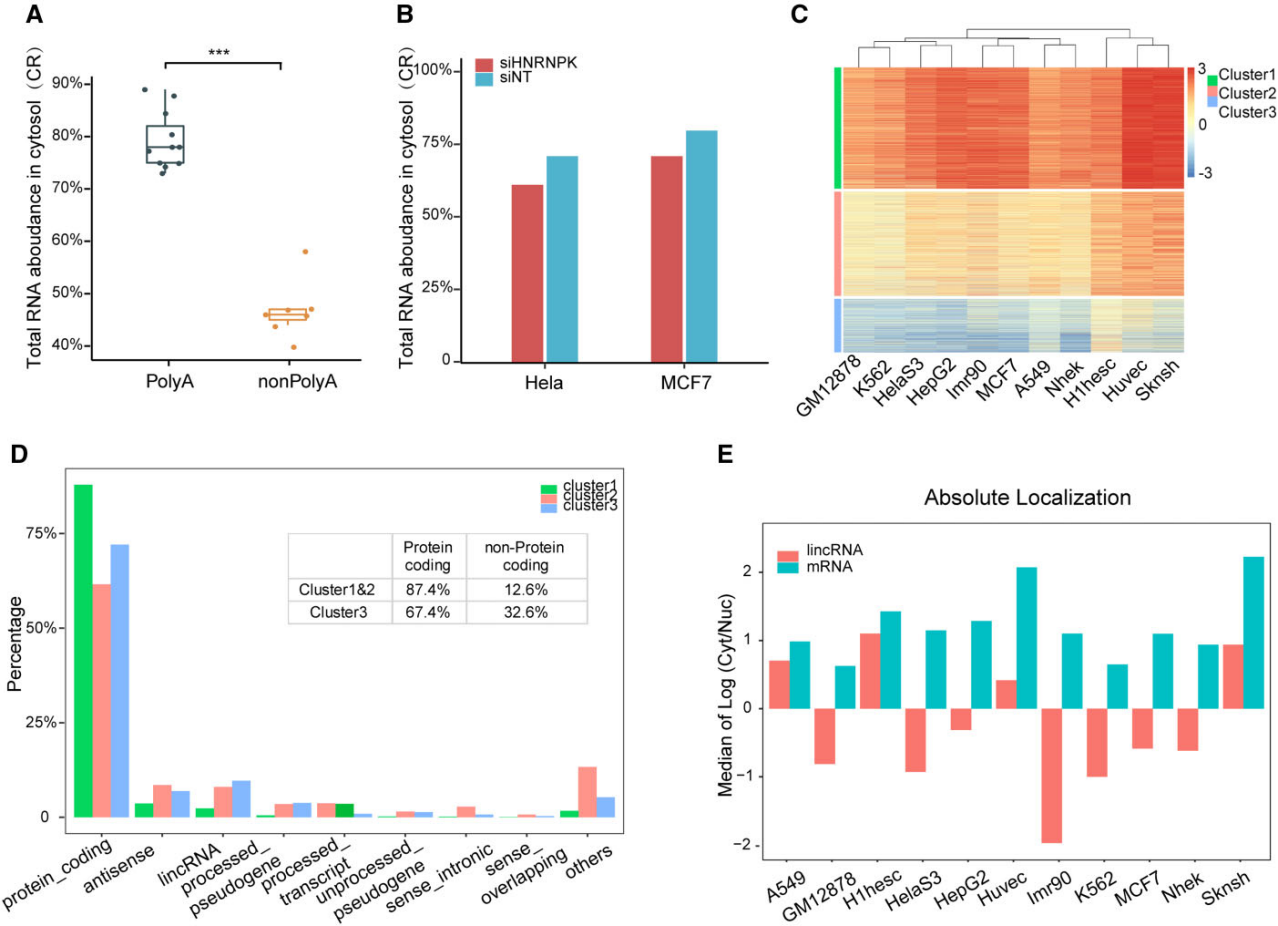
Application of SFAE to estimate CRs in multiple conditions. (A) Boxplot showing polyadenylated RNAs have significant higher CRs than nonpolyadenylated RNAs. The number (*n*) of cell lines analyzed is indicated. Statistical significance was determined by Wilcoxon rank-sum test. (B) Bar plot showing changes in CRs for two cell lines following *HNRNPK* knockdown by siRNA. (C) Heatmap of Z-scores of log2 Cyto/Nuc ratios of all genes after adjusting with CRs estimated by SFAE for 11 cell lines. Genes were clustered into three groups by K-means clustering, lincRNA and unprocessed pseudogenes are over-represented in bottom cluster, whose genes are more enriched in the nucleus *vs* cytosol. (D) Bar plot showing the percentage of different classes of RNA in three clusters defined in C. (E) Absolute localization ratios were estimated by adjusting TPMs with CR ratios estimated by SFAE. Median Log2-transformed Cyto/Nuc ratios for the all lncRNAs and mRNAs in each cell line are shown.

We applied this method to quantify the relative cytosolic and nuclear portion for polyadenylated and nonpolyadenylated RNA in the ENCODE datasets (Tilgner *et al.* 2012). As expected, majority of polyadenylated RNAs resided at cytosol, while nonadenylated RNAs did not show strong enrichment at cytosol (Figure  2A and Supplementary Figure S1 and Table S1). CRs of polyadenylated RNAs in human cell lines from different tissues were tightly distributed between ∼70% and 90%. Similar results were obtained in non-ENCODE studies (control in Figure  2B). The multiple fractionation techniques (CeFra-Seq) yielded similar CR estimate (Supplementary Table S1; Benoit Bouvrette *et al.* 2018). Similar levels of CRs were observed in D17 cell line from fruit fly (Supplementary Table S1; Benoit Bouvrette *et al.* 2018). Such estimates of CRs are also consistent with results (∼84%) from one single-cell study on human K562 cell lines (Abdelmoez *et al.* 2018). In addition, a study on mouse cell line using RNA-Seq and RNA-FISH showed that most transcripts have a 3.8 cytoplasm/nucleus ratio (Halpern *et al.* 2015), which was also consistent with the ∼70–90% CR ratios. In summary, all these evidences suggested the CRs predicted based on our method were consistent with results from existing studies. And such CRs suggested that using relative ratios between nuclear and cytosolic RNAs might underestimate the genes with an absolute abundance level preference for cytoplasmic localization.

### Altered relative RNA abundance of nuclear and cytosolic RNA upon perturbations

To understand whether overall abundance ratios between nuclear and cytosolic RNAs can be regulated, we looked into the subcellular fractionation RNA-seq dataset where perturbation is available. We observed a consistent decrease (∼10%) in cytosolic RNA ratios upon HNRNPK knockdown in two cell lines (Figure  2B). The decrease in cytosolic RNA also agreed with a larger number of nuclear-enriched genes (397) *vs* cytosol-enriched genes (283) upon perturbation (Lubelsky and Ulitsky 2018).

Unfortunately, such dataset is quite limited. Since we did not have a positive-control treatment that is known to alter the cytosolic RNA ratios, we resorted to ActD treatment, which inhibits transcription. Originally, we suspected that such cytosolic ratios (CRs) might increase as nuclear transcription was shut down. Both ActD inhibition (Supplementary Figure S2A) and RNA subcellular separation (Supplementary Figure S2B) were successful. However, CRs actually decreased slightly (from 0.629 in DMSO to 0.575 in ActD treatment) based on SFAE. We suspected that RNA degradation might play crucial roles in such process. Therefore, we extracted the short and long half-lives RNA in Hela cells from previous study (Sharma *et al.* 2016). Indeed, RNA with short half-lives exhibited a much stronger decrease in expression levels (Supplementary Figure S2C). Consistently, the RNAs with shorter half-lives showed a significant decrease in CRs, but not the RNAs with long half-lives (Supplementary Figure S2D).

Therefore, both examples above serve as a proof-of-principle that global cytosol ratios can be altered; further studies are needed to characterize how common this global CR is regulated under other conditions and biological processes.

### Majority of polyadenylated RNAs are preferentially located in the cytosol

As we obtained the correct estimates between the overall RNA abundances in cytosol and nucleus, we could calculate the absolute ratio between cytosol and nuclear localized mRNAs for each gene. We calculated such ratios for the polyadenylated mRNAs in ENCODE datasets, and clustered on both the gene and sample levels (Figure  2C). Consistently, majority of genes showed more enrichment in the cytosol *vs* nucleus. There were three major gene clusters based on K-means clustering algorithm, and the cluster in the bottom showed clear preference for nuclear localization pattern. As expected, nonprotein coding genes, such as lncRNA and antisense RNAs, were significantly enriched (*P*-value < 2.2e-16 based on a Chi-Square test) in this nuclear localized cluster (Figure  2D). Therefore, after adjusting with absolute CR ratio predicted by our method, the gene level localization patterns on the mRNAs (predominantly cytosol) and lncRNAs (predominantly nucleus) were also consistent with existing results (Chen and Carmichael 2009; Khalil *et al.* 2009; Derrien *et al.* 2012).

When focusing on the cell line specific patterns, we plotted the median log2 ratios between the absolute Cytosol *vs* Nuc TPM for mRNAs and lncRNAs as used in published literature (Figure  2E;Carlevaro-Fita and Johnson 2019). We also compared the results for 9 cell lines based on SFAE and multiple linear regression methods. Both methods predicted preferred localization in cytoplasm for mRNAs in all 9 cell lines. For lncRNAs, 5 out 9 cell lines showed consistent results (>50% of lncRNA located more in nucleus in HelaS3, IMR90, MCF7 cell lines, while >50% of lncRNA located more in the cytosol in SKNSH and HUVEC cell lines). For the other four cell lines (GM12878, HepG2, K562, and NHEK), SFAE predicted that >50% lncRNAs preferentially located in the nucleus, while multiple linear regression projected the opposite pattern (preference in cytoplasm). To eliminate potential bias caused by subtle differences in TPM and FPKM normalization method, we rerun the SFAE with FPKM as the expression input and obtained almost identical results (Supplementary Figure S3). We also calculated the error between predicted WC TPM and observed WC TPM by summing up the log ratios of all lncRNAs for two methods. They showed overall comparable levels of errors defined in Equation (1) (Supplementary Table S2). For the four cell lines showing inconsistent in overall pattern, two cell lines showed lower error in SFAE, while the other two cell lines showed lower errors in multiple regression based method. Thus, the error between estimated and observed WC TPM could not be used to determine which method is more accurate. The predicted output of SFAE was more consistent with prevalent view of nuclear localization of lncRNAs. However, lack of ground truth, we were unable to decide for sure which method yielded the correct estimates.

### Three gene classes based on the distribution pattern of polyadenylated and nonpolyadenylated RNAs

**Figure 3:**
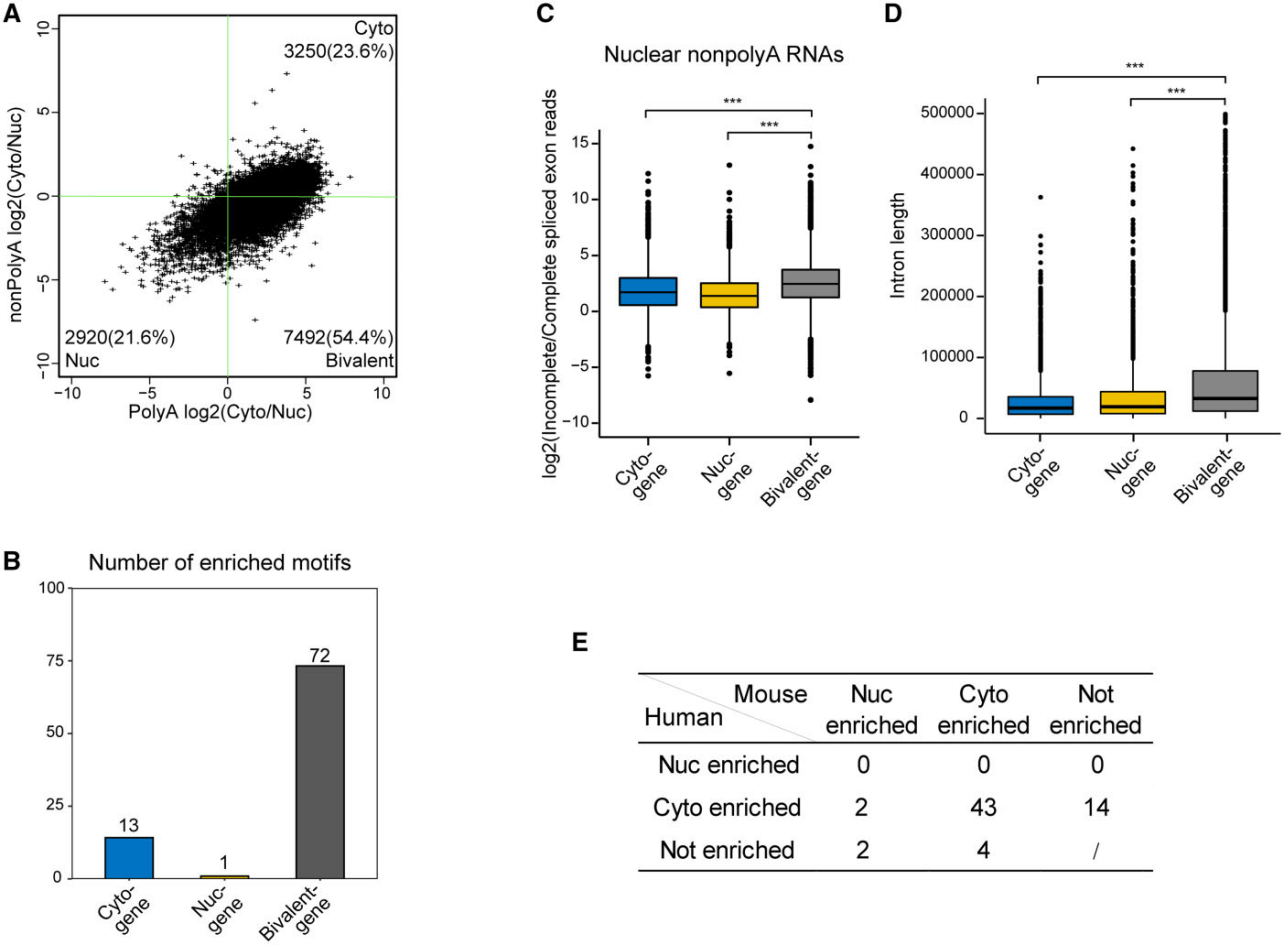
Three gene subgroups based on the distribution pattern of polyadenylated and nonpolyadenylated RNAs. (A) Scatter plot indicating three major group genes with based on median log2(Cyto/Nuc) ratios of polyA and non-PolyA data of cell lines. Genes on top-right and bottom-left are defined as Cyto genes and Nuc genes, as they are enriched in cytosol or nucleus irrespective of polyA tail status. Genes on bottom-right are defined as bivalent genes, which show distinct pattern based on polyA tail status. (B) Bar chart shows the number of enriched RBP motifs in the three gene clusters. (C) Boxplot shows log-transformed Incomplete/Complete spliced exon reads ratios of three gene clusters in nucleus for non-PolyA RNAs. *** represents *P*-value < 0.001 based on Wilcoxon rank-sum test for C and D. (D) Boxplot shows the summed intron lengths per gene distribution of three gene clusters. (E) Table shows the number of enriched RBP motifs with same or different localization preferences between human and mouse. Bivalent enriched motifs were considered as cytosol enriched motifs in here.

Most of studies on localization focused on polyadenylated RNAs, we also examined whether the localization patterns were consistent between RNAs with different polyA tail statuses. We quantified the median ratios across multiple cell lines between cytosolic and nuclear polyadenylated and nonadenylated RNA for each gene. Genes fell into three major categories (Figure  3A), and we defined three groups as nuclear, cytosolic, and bivalent genesets. Genes in cytosolic and nuclear subgroups showed consistent localization patterns irrespective of poly-adenylation status of RNAs, while genes in the bivalent group switched localization preferences when poly-adenylation status of mRNA changed: transcripts with polyA tails preferentially localized in the cytosol, while transcripts without polyA tails in the nucleus.

Previous studies suggested that *cis*-elements on RNA carried localization signals (Jambhekar and Derisi 2007; Bergalet and Lecuyer 2014; Chen 2016). Therefore, we extracted the Ensemble cDNA sequences for all genes and predicted the presence of annotated motifs recognized by RNA binding proteins (RBPs) (Ray *et al.* 2013). We identified the enriched motifs based on their motif densities in each geneset (see *Materials and* *Methods* for more details). Interestingly, compared with other two groups, genes in nucleus group only have a much smaller number of enriched RBP motifs (Figure  3B and Supplementary Table S3), suggesting that their transcripts might contain fewer cytosolic export *cis*-elements. *RBM4*, which is the most enriched RBP in the cytosolic genes, contains a C-terminal alanine-rich domain that potentially mediate RNA export (Lai *et al.* 2003). RNA export function of *SRSF1*, another top enriched RBP, is also supported by a few published literatures (Das and Krainer 2014; Khan *et al.* 2021). However, the functional roles of RBP in RNA localization, such as RNA export, were still very limited. Therefore, we were unable to confirm whether these enriched motifs we predicted for cytosolic genes are indeed functional *cis*-elements. Our study provided a prioritized list for mechanistic studies.

However, the localization pattern of bivalent genes could not be simply explained by *cis*-elements. We argue there are at least two models to explain the distinct localization preference based on polyA tail status. One possibility is that for these genes, a tight quality control was implemented and only fully processed transcripts with polyA tail were exported. As previous studies reported that RNA export is coupled with RNA processing, such as RNA splicing (Luo and Reed 1999; Zhou *et al.* 2000). To validate such assumption, we quantified the ratio between reads from incompletely spliced transcripts (reads with fragments containing intron) *vs* reads from fully spliced transcripts (reads only containing exons) for all genes in nuclear and cytosol subcellular fractions. Indeed, such ratios in bivalent genes were significantly higher compared with other two types for nuclear RNAs in nonpolyadenylated RNAs (Figure  3C). Interestingly, similar pattern was also observed in the nuclear RNAs with polyA tails (Supplementary Figure S4A), but not in the cytosolic RNAs with or without polyA tails (Supplementary Figure S4, B and C). To understand whether the longer total intronic length in polyA-dependent group led to higher ratios of incomplete splicing, we quantified the total intronic length for each gene in three groups. Indeed, the total intron length in bivalent genes were significantly longer, which might lead to a slower processing rate and higher fraction of incompletely processed transcripts (Figure  3D). This suggested that significant higher proportion of nonpolyadenylated RNAs from bivalent genes may be immature transcripts and thus be retained in the nucleus until the completion of RNA splicing.

Another alternative explanation is that both polyA and non-PolyA transcripts had similar localization preference. But cytosolic poly-adenylation altered the pattern (see cartoon model in Supplementary Figure S5). Indeed, only bivalent genes, but not cytosolic genes, showed strong enrichment with cytoplasmic polyadenylation related genes such as *CPEB2-4* (Supplementary Table S3; Charlesworth *et al.* 2013).

To understand whether these RBP mediated localization are conserved between species, we also processed the subcellular fractionation data from mouse. Indeed, we observed highly consistent pattern of enrichment in the same subcellular fractionation (Figure  3E and Supplementary Table S4). For instance, *CPEB* motifs were enriched in cytosolic genes in both species. Conserved RBP mediated localization between species is consistent with that CPEBs are cytoplasmic polyadenylation element-binding proteins (Novoa *et al.* 2010; Charlesworth *et al.* 2013).

### Variations of distribution pattern in different cell lines

**Figure 4:**
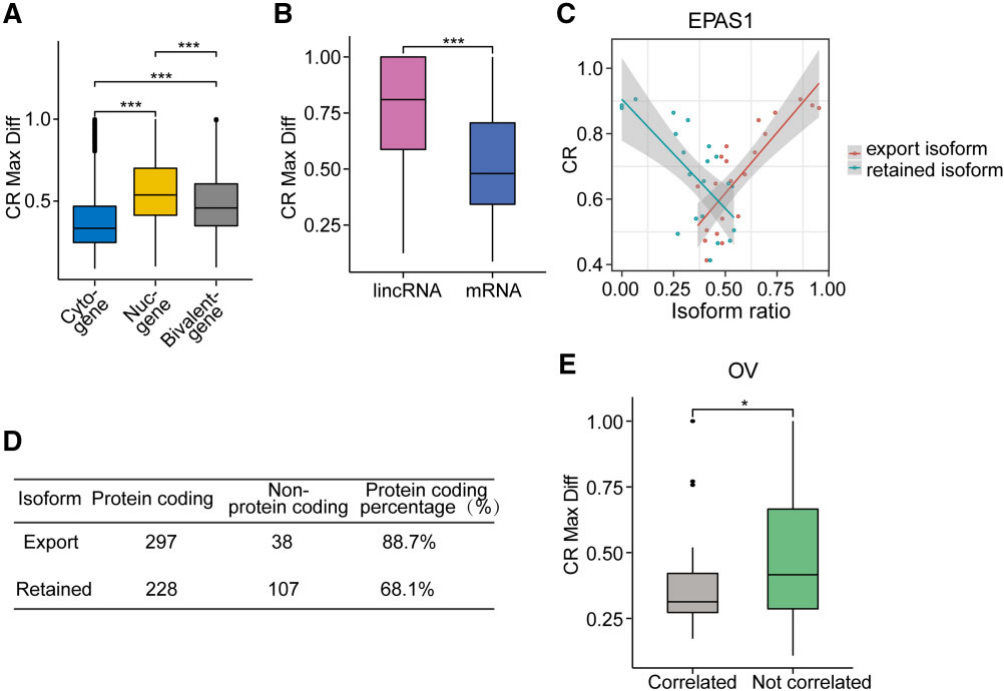
Variations of CR in multiple cell lines on the gene level. (A) Boxplot shows CR maximal differences (CR Max Diff) on individual gene level among different cell lines in three defined gene clusters. Genes in different groups showed significant differences in the CR Max Diff based on Wilcoxon rank-sum test for this and other panels in this figure if not mentioned otherwise. (B) Boxplot shows lincRNA have significantly higher CR variations based on CR Max diff than mRNA. (C) Correlation between CR and isoform ratios of *EPAS1* gene. Each circle represents one cell line. Two highest expressed isoform showed opposite correlation pattern with CRs. (D) Annotation of transcript types (protein coding or nonprotein coding) on export and retained isoform. (E) CR Max Differences between two groups genes in TCGA ovarian cancer dataset. Correlated and Not correlated, respectively, were defined based on whether genes showing and not showing significant positive Pearson correlation between mRNA and proteins from ovarian cancer CPTAC projects.

Only the average value across multiple cell lines were shown in Figure  3A, however, the distribution preference might differ for the same gene in different cell lines. Therefore, we quantified the variations in the form of maximum difference among all the cell lines and variance of the CR ratios. Nuclear genes showed the largest variations, while cytosolic genes showed least variations for polyadenylated RNAs (Figure  4A). Similar pattern was observed when the variances of CR ratios were plotted (Supplementary Figure S6A). Given general localization preference in the nucleus for lncRNA and cytosol for mRNAs, we quantified the variations of distribution for these two types of genes. Consistently, genes encoded mRNA showed significant lower variation in cellular distribution preferences (Figure  4B and Supplementary Figure S6B).

Previous study showed that alternative isoforms exhibited different localization patterns (Taliaferro *et al.* 2016), therefore we wanted to evaluate whether alternative isoform splicing may explain the variations in localization in different conditions. We used RSEM to quantify the isoform level expression for all the genes. And we also calculated the Pearson’s correlation coefficient between the CRs values and isoform expression levels for all 11 cell lines. Then we looked for genes whose two highest expressed isoforms showed opposite correlation with CRs. In total, 335 genes with a relative high expression level (median TPM ≥ 3) showed such pattern (see one example on gene EPAS1 in Figure  4C and Supplementary Table S5). We examined the transcript categories for the exported (cytosolic) and retained (nuclear) isoforms based on their correlation with CR s. Indeed, the exported isoforms were significantly more enriched with protein-coding isoforms (*P*-value ≤ 9.42e-11 based on Fisher’s Exact test, Figure  4D).

### Variations of RNA distribution severely affects transcription process in cancers

Given the potential mechanism of regulating localization to control protein production, we suspected that the part of genes exhibited lower mRNA-protein correlation might adopt localization-based regulatory mechanisms. Therefore, we extracted the genes showing and not showing significant positive correlation between mRNA and proteins from breast cancer and ovarian cancer in CPTAC projects (Edwards *et al.* 2015). Indeed, genes with strong mRNA-protein correlation showed significant lower levels of variations in term of CRs in both datasets (Figure  4E and Supplementary Figure S6C), suggesting controlling mRNA export can be a general mechanism to regulate translation.

## Discussion

Given the complexity to correctly estimate relative total transcript abundances in different cellular fractions, most of the existing studies adopted relative localization ratios for comparison (such as cytosol/nucleus ratios). Cyto/Nuc Ratios in polyadenylated RNAs predicted by our method showed clear imbalance between cytosolic and nuclear RNAs: there are consistently more RNAs resided in the cytoplasm compared with nuclear for all cell lines and conditions (Figure  2A). In addition, we showed that such Cyto/Nuc ratio could vary between different RNA subtypes, cell lines, or different conditions from the same cell line (Figure  2). The dynamic regulation of nuclear localization can occur on multiple levels. First, the global RNA abundance Cyto/Nuc ratio can change as we have observed in the HNRNPK knockdown and ActD treatment experiments. Interestingly, ActD treatment did not increase in CR upon transcription shutdown in the nucleus as expected, possibly due to the decrease of polyA mRNA in the cytosol caused by mRNA degradation was stronger than the decrease of polyA mRNA production in the nucleus due to the transcription shutdown of ActD treatment (Tatosyan *et al.* 2020). It will be interesting to explore whether during disease states or developmental stages, such global nuclear/cytosol RNA ratio could be altered. If so, whether this regulation has functional consequences and its regulatory mechanism.

We also showed that the localization pattern for each gene transcript could be dynamically regulated. For the genes exhibited large variations, we identified alternative isoforms that may exhibit distinct localization patterns. Therefore, by altering the relative abundance of different isoforms, the localization pattern can be switched. This is consistent with previous report that during neuronal differentiation, isoform switching occurs to regulate mRNA localization (Taliaferro *et al.* 2016). A cell-type-specific stability regulation might also explain the localization patterns for different isoforms in cell lines. Previous studies showed the stability of transcripts in different cell types or the same cell type in different conditions could be tuned in an isoform specific manner (Zhang *et al.* 2002; Tushev *et al.* 2018). Nevertheless, we suspected that localization regulation could be adopted to control translation. Consistently, the cytosol localizing isoforms were enriched with protein-coding isoforms (Figure  4D). In addition, we also observed that the genes with high mRNA-protein levels concordance from CPTAC datasets showed weaker variations in localization patterns (Figure  4E).

There are a number of possible regulatory mechanisms for RNA localization. By integrating analyses on non-PolyA and polyA RNAs, we identified a large polyA-dependent cytosolic geneset whose localization patterns differ based on polyadenylation tail status (Figure  3A). Previous studies showed the RNA export is frequently coupled with splicing and RNA maturation (Luo and Reed 1999; Zhou *et al.* 2000). Therefore, it is also possible that polyA tail just reflected the completion of RNA processing, which is coupled with RNA export. In addition, we identified the RBP motifs enriched with gene groups with distinct localization pattern. The motifs we identified were consistent with existing studies and also across species. We did observe that several RBP motifs showed opposite patterns in localization with respect to preference in nuclear or cytosolic fractions. A recent study showed that the difference in abundance of RBP protein levels, instead of *cis*-elements sequences on lncRNA, lead to opposite localization patterns between human and mouse embryonic stem cells (Guo *et al.* 2020). Due to the functional and structure conservation for the same RBPs, we suspected that the different regulation of abundance for these RBPs across species might be more likely cause for opposite localization patterns, instead of distinct localization patterns dictated by the homologous RBP genes in two closely related species. However, one limitation of our approach is that we relied on curated motif database, which might not truly reflect the true complexity of *cis*-elements that regulate the localization.

The other limitation with our work is lack of enough biological replicates. Due to the small number of subcellular fractionation RNA-Seq, most of the sample we only have one or two biological replicates. Therefore, we are unable to dissect the variations caused by different cell lines and intrinsic variations within one cell line. On the global level, we did observe similar CR ratios for MCF7 and Hela cell lines generated from different labs (Figure  2 and Supplementary Table S1). It will be interesting to estimate the intrinsic variations of genes on RNA localization when no other biological variables were present, which will facilitate the identification of changes in localization caused by perturbations or different biological conditions (such as cell type) instead of intrinsic variations.

In summary, we presented a SFAE method to estimate relative ratios for multiple subcellular fractions. By applying our methods, we were able to show the variations in RNA localization from multiple levels. Our results suggested that RNA localization could be dynamically regulated, therefore, it will be interesting to explore the functional consequences (such as the translational control) and significance of such regulated RNA localization in multiple physiological processes and disease conditions.

## Data availability

The raw sequence data reported in this paper have been deposited in the Genome Sequence Archive (Chen *et al.* 2021) in National Genomics Data Center (CNCB-NGDC Members and Partners 2021), China National Center for Bioinformation/Beijing Institute of Genomics, Chinese Academy of Sciences (GSA: HRA001427) that are publicly accessible at https://ngdc.cncb.ac.cn/gsa.

SFAE method is available as a standalone Python pipeline (github.com/bioliyezhang/SFAE).

Supplementary material is available at *G3* online.

## Supplementary Material

jkab371_Supplementary_Figures

jkab371_Supplementary_TableS1

jkab371_Supplementary_TableS2

jkab371_Supplementary_TableS3

jkab371_Supplementary_TableS4

jkab371_Supplementary_TableS5
