# Supplementary material for: Application of RNA subcellular fraction estimation method to explore RNA localization regulation
Source: G3 (Bethesda). 2021 Nov 13;12(1):jkab371. doi: 10.1093/g3journal/jkab371 (PMC8727992; doi:10.1093/g3journal/jkab371)
Supplement: jkab371_Supplementary_Figures [file jkab371_supplementary_figures.docx]

***Supplementary Materia****l*

**Supplementary Figures:**

**
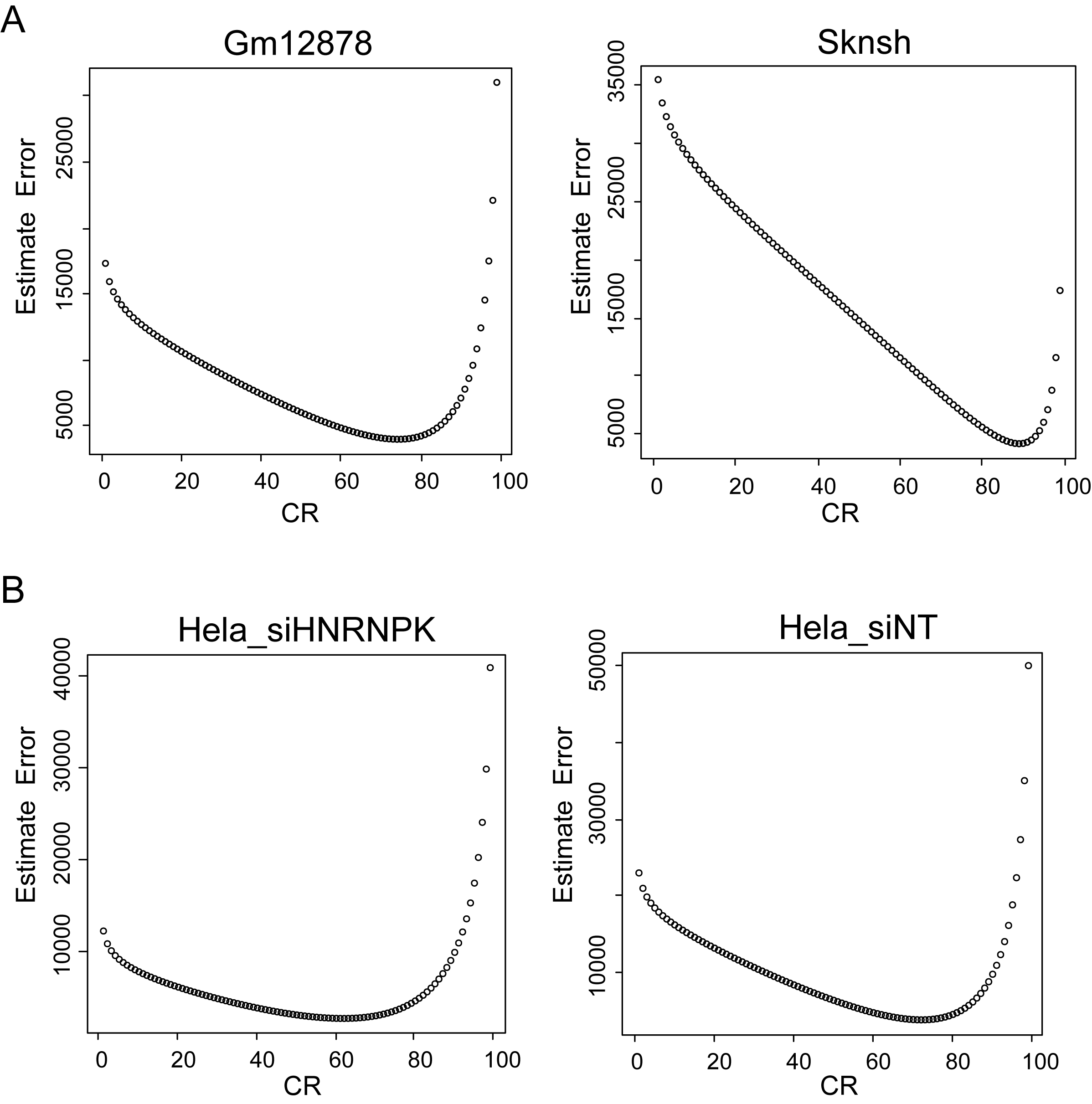
**

**Figure S1**. Cytosolic RNA abundance Ratios (CRs) dependent Errors distribution. (A) The curves of two cell lines in 11 cells lines show estimated errors corresponding to different CRs from 0 to 1. (B) Similarly, estimate errors curves for HNRNPK perturbation and Control in Hela cell line are plotted.


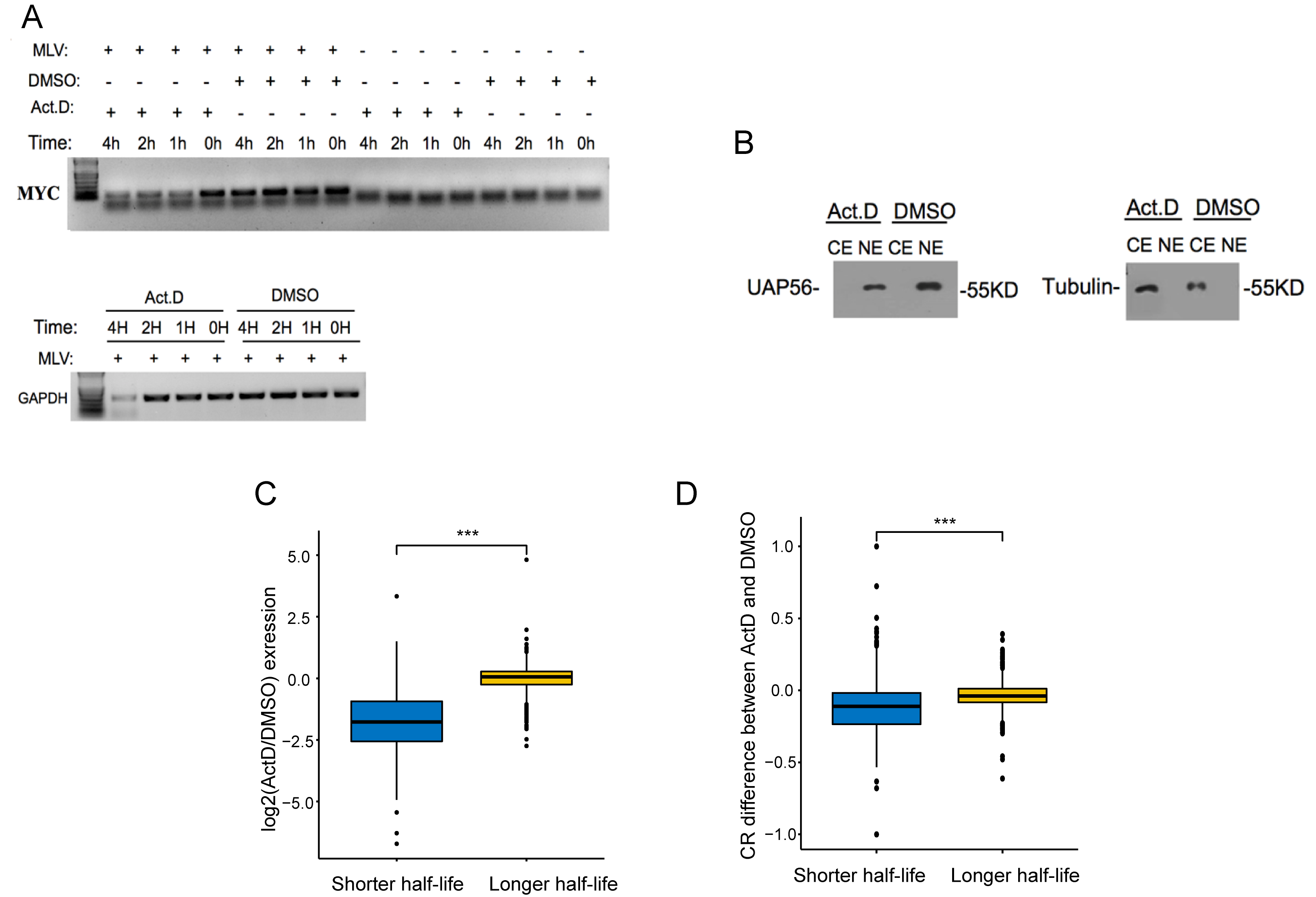


**Figure S2:** Expression and CR change upon ActD treatment. (A) RT-PCR of representative genes (Above) MYC and (Below) GADPH upon ActD treatment. MLV stands for MLVRTase, is a reverse transcriptase. (B) The Western blot of representative nuclear and cytosol localized proteins in separated nuclear and cytosolic fractions. (C) Log fold change of ActD and DMSO treatment whole cell expression level between genes of shorter half-lives and longer half-lives. Shorter half-life represents genes whose half-life less than 2 hours, longer half-life represents genes whose half-life more than 6 hours. (D) CR difference between ActD and DMSO treatment. CR difference was defined as CR_ActD_-CR_DMSO_. Wilcoxon rank-sum test was performed to evaluate whether there is significant difference between two groups in (C) and (D). *** represents P value smaller than < 0.001


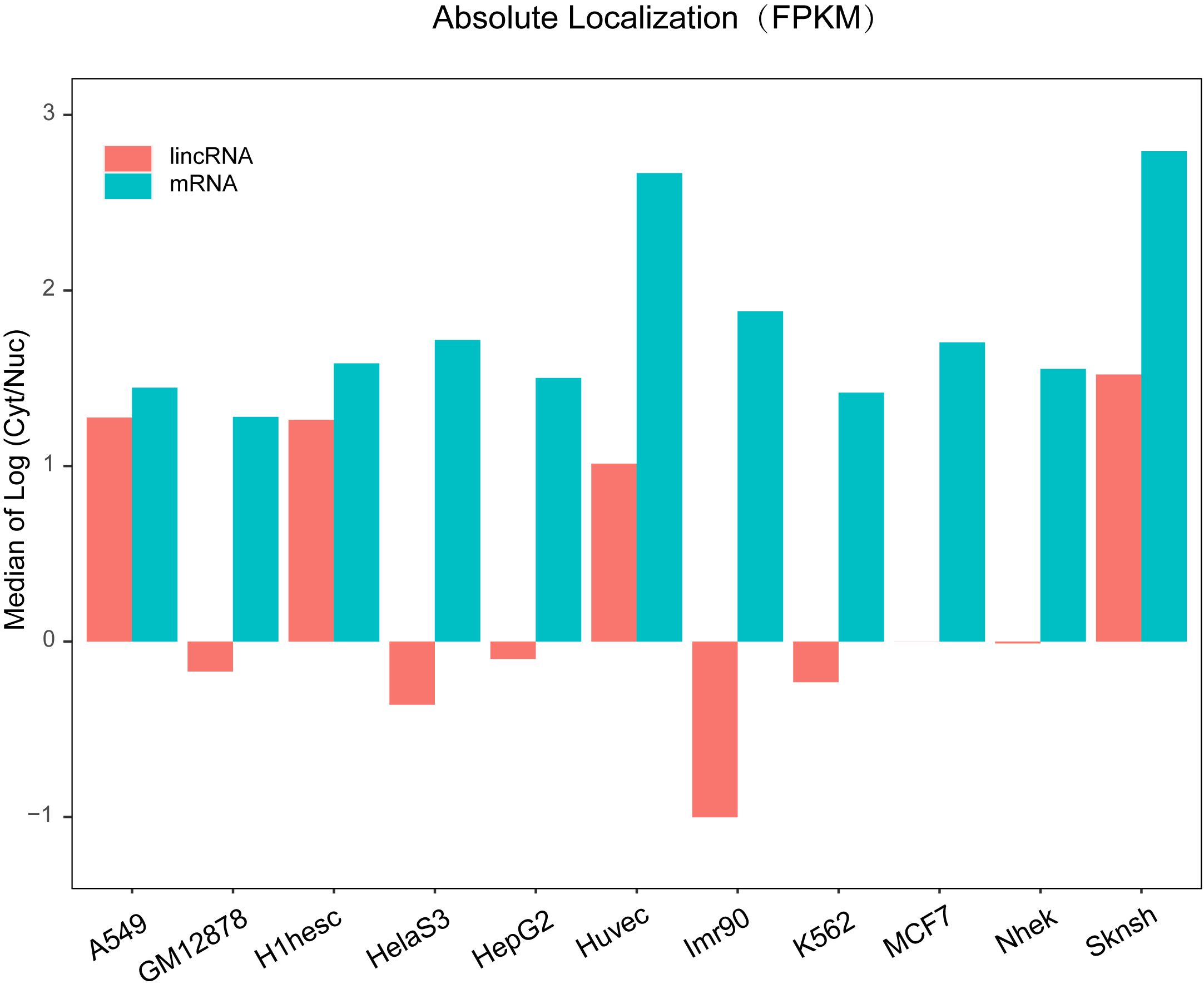


**Figure S3.** Absolute localization ratios were estimated by adjusting FPKMs with CR ratios estimated by SFAE. Median Log2-transformed Cyto/Nuc ratios for the all lncRNAs and mRNAs in each cell line are shown.


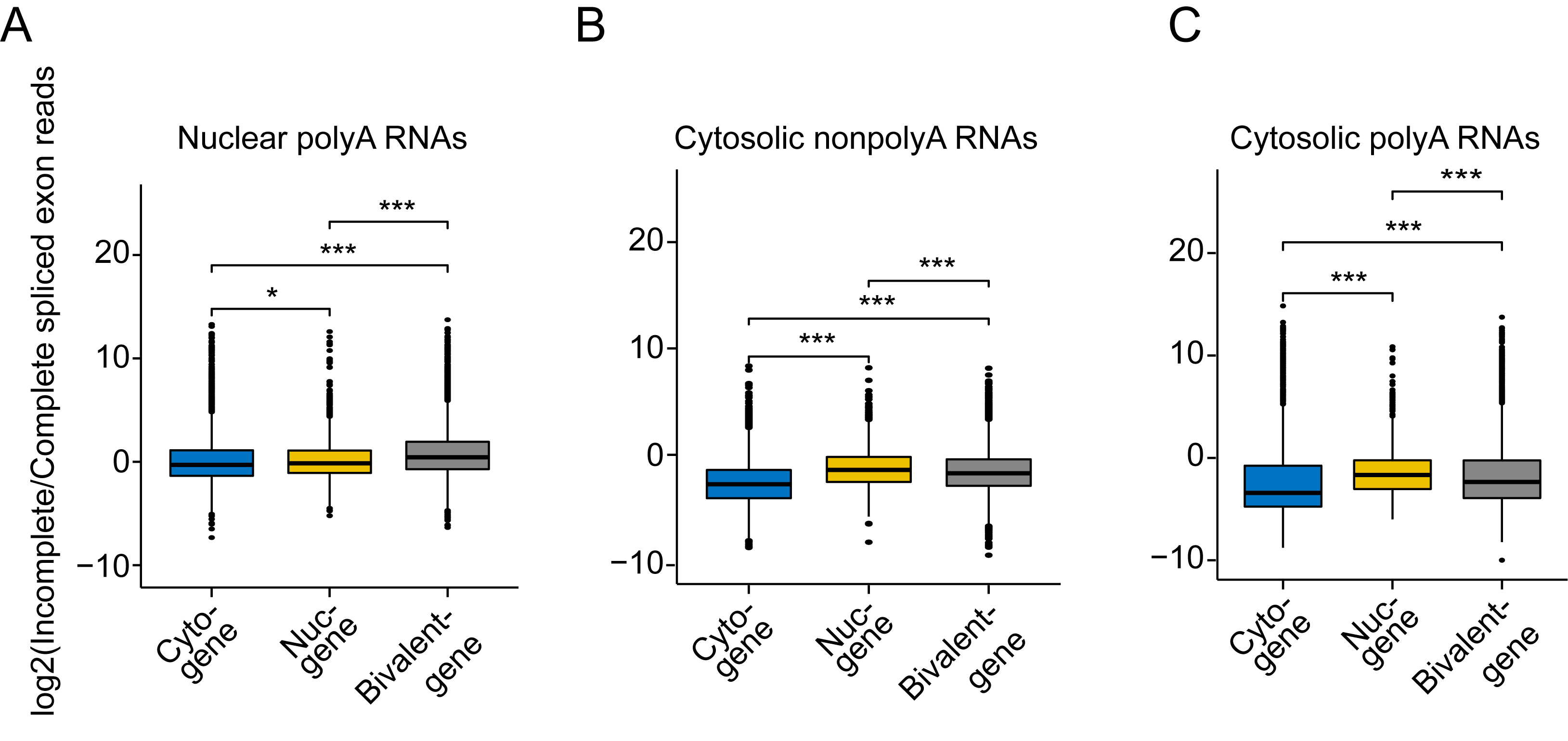


**Figure S4**. Incomplete vs completed splicing ratios in different RNAs population. Boxplot shows log-transformed Incomplete/Complete spliced exon reads ratios of three gene clusters in nuclear PolyA RNAs(A); in cytosolic nonPolyA RNAs (B) and in cytosolic PolyA RNAs(C). Wilcoxon rank-sum test was performed to evaluate whether there is significant difference between two groups. *** represents P value smaller than < 0.001.


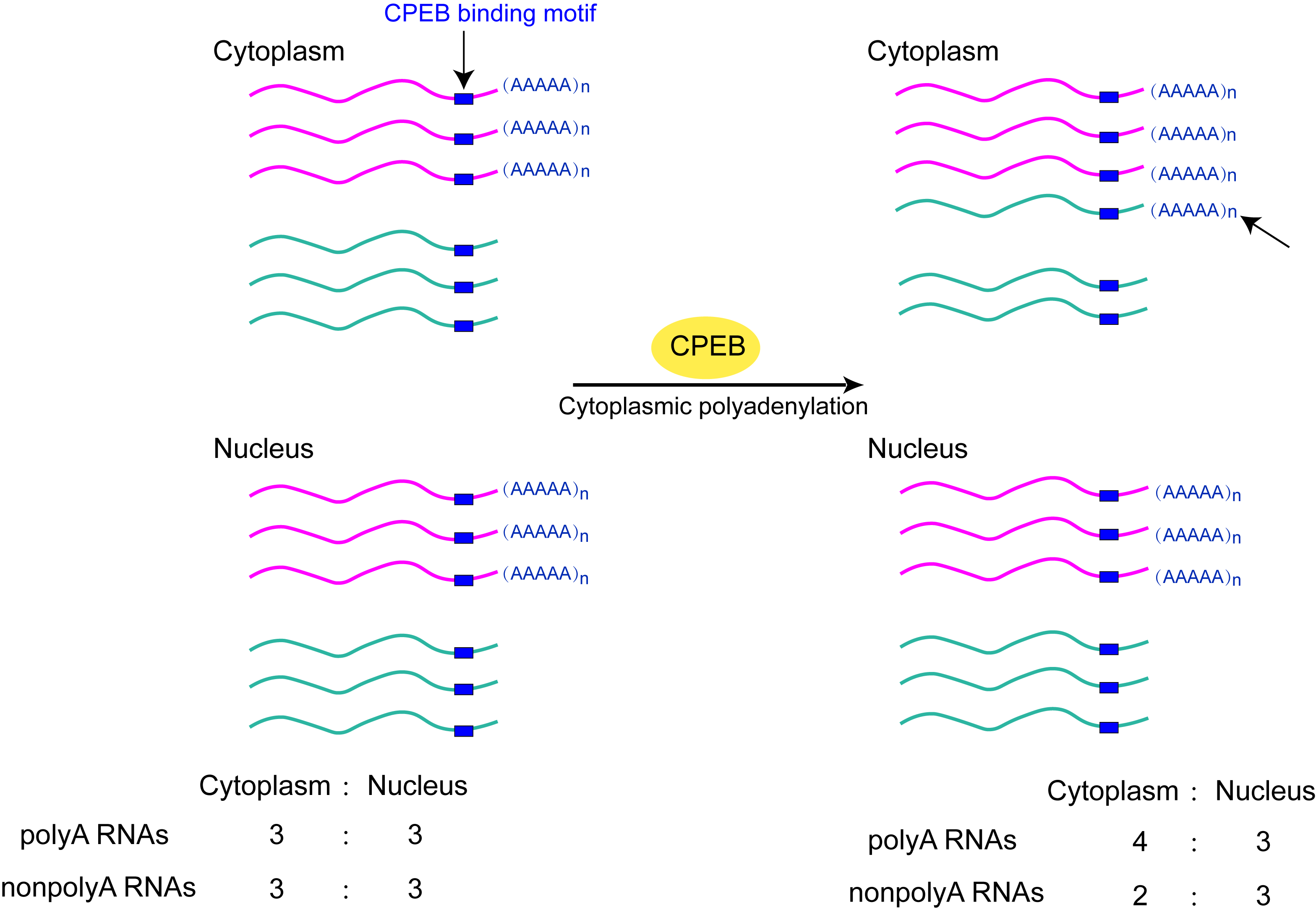


**Figure S5**. Model for opposite localization preferences for bivalent genes with and without polyA tails with CPEB motifs. Carton figure shows the increase of polyadenylated RNAs and decrease of non-polyadenylated RNA can occur in the cytosol, but not in the nucleus, by CPEB mediated cytosolic polyadenylation. This eventually may lead to opposite localization patterns as showed in the example data on the bottom of the figure.


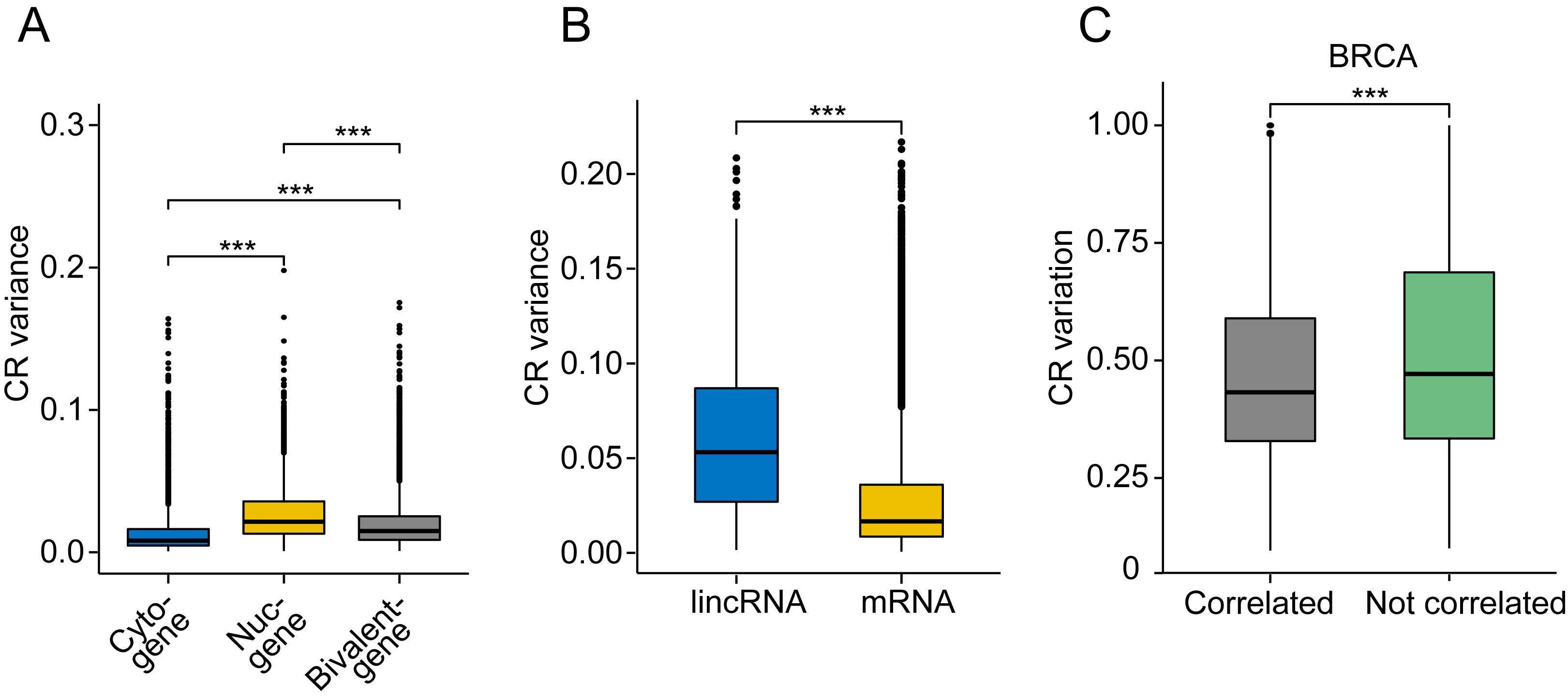


**Figure S6**. Variances of CR in multiple cell lines on the gene level. (A) Boxplot shows CR variances on individual gene level among different cell lines in three defined gene clusters. Genes in different groups showed significant differences on the variances. (B) Boxplot shows lincRNA have significantly higher CR variances than mRNA. (C) CR maximal differences between two groups genes in TCGA breast cancer dataset. Correlated and Not correlated, respectively, were defined based on whether genes showing and not showing significant positive correlation between mRNA and proteins from breast cancer CPTAC projects, similar to Figure 4E. All the statistical test was performed based on Wilcoxon rank-sum test.
